# Sicker patients account for the weekend mortality effect among adult emergency admissions to a large hospital trust

**DOI:** 10.1136/bmjqs-2018-008219

**Published:** 2018-10-09

**Authors:** Jianxia Sun, Alan J Girling, Cassie Aldridge, Felicity Evison, Chris Beet, Amunpreet Boyal, Gavin Rudge, Richard J Lilford, Julian Bion

**Affiliations:** 1 Health Informatics, University Hospitals Birmingham NHS Foundation Trust, Birmingham, UK; 2 Institute of Applied Health Research, University of Birmingham, Birmingham, UK; 3 Intensive Care, University Hospitals Coventry and Warwickshire NHS Trust, Coventry, UK; 4 Research & Development, University Hospitals Birmingham NHS Foundation Trust, Birmingham, UK; 5 Public Health, University of Warwick, Coventry, UK; 6 Intensive Care Medicine, University of Birmingham, Birmingham, UK

**Keywords:** mortality (standardized mortality ratios), patient safety, duty hours/work hours, emergency department, hospital medicine

## Abstract

**Objective:**

To determine whether the higher weekend admission mortality risk is attributable to increased severity of illness.

**Design:**

Retrospective analysis of 4 years weekend and weekday adult emergency admissions to a university teaching hospital in England.

**Outcome measures:**

30-day postadmission weekend:weekday mortality ratios adjusted for severity of illness (baseline National Early Warning Score (NEWS)), routes of admission to hospital, transfer to the intensive care unit (ICU) and demographics.

**Results:**

Despite similar emergency department daily attendance rates, fewer patients were admitted on weekends (mean admission rate 91/day vs 120/day) because of fewer general practitioner referrals. Weekend admissions were sicker than weekday (mean NEWS 1.8 vs 1.7, p=0.008), more likely to undergo transfer to ICU within 24 hours (4.2% vs 3.0%), spent longer in hospital (median 3 days vs 2 days) and less likely to experience same-day discharge (17.2% vs 21.9%) (all p values <0.001).

The crude 30-day postadmission mortality ratio for weekend admission (OR=1.13; 95% CI 1.08 to 1.19) was attenuated using standard adjustment (OR=1.11; 95% CI 1.05 to 1.17). In patients for whom NEWS values were available (90%), the crude OR (1.07; 95% CI 1.01 to 1.13) was not affected with standard adjustment. Adjustment using NEWS alone nullified the weekend effect (OR=1.02; 0.96–1.08).

NEWS completion rates were higher on weekends (91.7%) than weekdays (89.5%). Missing NEWS was associated with direct transfer to intensive care bypassing electronic data capture. Missing NEWS in non-ICU weekend patients was associated with a higher mortality and fewer same-day discharges than weekdays.

**Conclusions:**

Patients admitted to hospital on weekends are sicker than those admitted on weekdays. The cause of the weekend effect may lie in community services.

## Introduction

Since Bell and Redelmeier first reported weekend admissions associated with significantly higher mortality rates for 23 of the 100 leading causes of death in Canada in 2001,^1^ many studies around the world have also shown the so-called ‘weekend effect’.^2–10^ The phenomenon has been observed in both emergency and elective admissions and in different healthcare systems. Causation was for many years attributed to suboptimal hospital staffing on weekends, particularly senior medical staffing, and this has been used to support government policies such as ‘seven day services’.^11 12^ However, little evidence has been adduced to support this hypothesis. In theory reduced weekend staffing and resources should affect all hospitalised patients, not just those newly admitted, but studies have shown a lower mortality rate among hospitalised patients on weekends compared with weekdays^4 5^ and the High-intensity Specialist Led Acute Care (HiSLAC) study found no relationship between intensity of specialist staffing and mortality among weekend admissions.^13^

More recently, Meacock *et al* have shown that on weekends there is a marked reduction in admissions of patients referred by community services,^14^ with indications that the fewer patients who were admitted on weekends were more severely ill (online supplementary ESM figure 1). Assessment of severity has conventionally been performed using variables such as demographic characteristics, primary diagnosis and comorbidity.^4–10^ While this approach is important, it fails to capture fluctuating components of illness, most particularly abnormal physiology. Physiology-based scoring systems add value to diagnosis-based systems^15 16^ since changes in physiological vital signs provide early warning of impending clinical deterioration, and can be used to calculate probability of in-hospital mortality. Standard vital signs have now been incorporated in the National Early Warning Score (NEWS) recommended by the Royal College of Physicians for tracking clinical deterioration and alerting the medical team to take preventative action.^17^ The physiological vital signs included in NEWS are respiration rate, oxygen saturation, temperature, systolic blood pressure, pulse rate, level of consciousness and whether or not supplemental oxygen was required (online supplementary ESM figure 2). NEWS is categorised in four bands representing different levels of clinical risk and the clinical responses required^17^ (online supplementary ESM figure 3).

10.1136/bmjqs-2018-008219.supp1 Supplementary data

Three studies have now examined this aspect of case mix. A single-centre study supplemented case mix adjustment with an acuity score based on laboratory variables and oxygenation to show that weekend admissions had more respiratory, renal and neurological diseases, and more biochemical derangements; however, they did not exclude the possibility that these effects were attributable to a reduction in weekend presentation by less severely ill patients.^18^ A study from four hospitals in a single trust used laboratory tests as the measure of severity of illness to show that the weekend effect was attributable to a marked reduction in admission of less severely ill patients on weekends and on public holidays compared with weekdays.^19^ The third study examined 48774 emergency admissions to four community hospitals and used electronically recorded vital signs to demonstrate greater severity of illness among weekend admissions.^20^ Admission rates were lower on weekends in this study, again suggesting the likelihood of fewer low-risk admissions on weekends. Adjustment for severity using the NEWS reduced the crude (unadjusted) weekend:weekday OR for death from 1.10 to 0.99, but their standard model only included age, sex and admission month. Mortality rates were substantially higher in patients for whom no NEWS was recorded, and NEWS completion rates were higher on weekends, but the authors did not explore the reasons for the missing NEWS. The authors emphasised the need for confirmatory studies incorporating information on patient pathways.

Using the electronic database of a large university teaching hospital, we have undertaken this work as part of the HiSLAC project (www.hislac.org) funded by the HS&DR programme to investigate the weekend effect and evaluate the roll-out of 7-day services. We have examined admission pathways, case mix and severity of illness (first complete NEWS) within the first 24 hours of admission.

## Methods

We have analysed 4 years of data from the electronic clinical databases of the Queen Elizabeth Hospital Birmingham, one of the largest single-site university teaching hospitals in England, caring for more than 800 000 patients every year and providing tertiary referral services across the UK. The hospital has 1213 inpatient beds, 32 operating theatres and a 100-bed unitary intensive care unit (ICU).^21^ A computerised MS SQL database supports a comprehensive clinical information system which includes administrative, diagnostic, physiological and laboratory data. At the time period of this study (2012–2015) vital signs of admitted patients were recorded electronically except in the ICU where the system was still in development: consequently, physiology was recorded manually on the ICU paper chart. We were therefore unable to calculate NEWS for the majority of patients transferred directly to the ICU from the emergency department (ED).

### Data extraction

We identified all adult emergency admissions over a 4-year period from January 2012 to December 2015. Before 2012 the electronic patient information system was still in the roll-out phase of implementation. We extracted basic demographic information (age, sex, ethnicity, deprivation, principal diagnosis (International Classification of Diseases 10th Revision), comorbidities) and outcome (survival at 30 days after admission and hospital discharge). Readmissions were classified as new hospital spells. Charlson Comorbidity Index was calculated and categorised according to the English NHS Summary Hospital-level Mortality Indicator (SHMI).^22^ Principal diagnosis was grouped using SHMI groupings. Income deprivation index was based on the 2015 Index of Multiple Deprivation data based on the patient’s residential postcode. The number of previous admissions within 30 days before the index admission was categorised as none, 1, 2 or ≥3. Admission source was extracted and classified as from usual residence and elsewhere. Admission routes have also been extracted and grouped as from Accident and Emergency, general practitioner (GP) and bed bureau, consultant clinic and other routes.

We supplemented these standard measures of case mix with information on whether or not patients had been transferred to the ICU, and data on patients’ vital signs, as specific indicators of severity. This involved linking and matching between separate databases. Transfer to ICU within 24 hours of hospital admission (‘ICU24’) was determined from ward transfer data and admission time; ICU transfers were classed as those direct from the ED, or indirect transfers via another in-hospital location following the initial admission. NEWS was calculated as defined in national guidance^17^ by using the first full set of vital signs available within 24 hours following admission; we have termed this ‘NEWS24’. NEWS was not calculated by the hospital staff, but from the primary variables in the database as defined.^17^ As described above, in the period during which these data were collected, the hospital had not yet installed electronic data capture for vital signs in patients admitted to intensive care; critically ill patients transferred directly from the ED to the ICU bypassed electronic capture of vital signs by the clinical information system. Therefore, the patients were classified into six risk categories using ICU status and NEWS24 values as follows: The (putative) highest risk category consisted of patients admitted to intensive care within 24 hours (ICU24). The remaining (non-ICU24) patients were assigned, where possible, to one of four NEWS severity bands given by NEWS24 ≥7; 5–6; 1–4; and 0. The sixth category consisted of those non-ICU24 patients for whom NEWS24 was missing.

Weekend admissions were defined as admitted on or after midnight on Friday until midnight on Sunday. Admission time was the point of admission into the hospital, not the time when the patient first presented to the ED. Length of stay (LOS) was calculated by subtracting day of admission from day of discharge. Admitted patients who were admitted and either discharged or died before midnight on the day of admission are classified as zero LOS (Z-LOS).

### Statistical analysis

Descriptive statistics for demographic data, routes of admission, ICU transfer, LOS and NEWS status included means/SDs, medians/IQRs, and percentages. Logistic regression methods were used to analyse mortality 30 days after admission. Mortality at hospital discharge is treated in appendices (online supplementary ESM tables 1 and 2). Outcome was linked to admissions. Where death occurred within 30 days of multiple admissions, the outcome was linked with each of the admissions and the number of previous admissions was included in the model, replicating the method used by Freemantle and colleagues.^4 5^ We used multivariable logistic regression to estimate the weekend (WE) to weekday (WD) OR for death following admission (WEOR). The ‘Standard model’ employed conventional risk adjustment variables (age—restricted cubic spline with five knots: sex, ethnicity, day of year, admission source, diagnostic category—SHMI grouping, income deprivation, number of preadmissions and comorbidity—Charlson Comorbidity Index category). Since the data set includes 4 years of data, calendar year is also included as an adjustment variable. This approach is similar to that of Freemantle and colleagues.^4 5^ Then a second set of models was developed incorporating patient risk strata (ICU24 and NEWS band) in the standard adjustment model, and the WEOR of each group was then calculated. Finally, we examined the effect of severity of illness on weekend-weekday admission mortality by adjusting crude mortality for NEWS alone. The data extraction and cleaning process was done in SQL. The statistical analyses were performed using Stata/SE V.14. P values are two sided.

## Results

### Population and pathway characteristics

#### Demographics

A total of 163 134 emergency admissions were recorded on the trust’s clinical information system between 1 January 2012 and 31 December 2015. One patient with unrecorded sex and five patients younger than 16 years of age were excluded, providing 163 128 emergency admissions for analysis. Median age was 60 years and did not vary by day of the week. Overall there were no differences between weekend and weekday admitted populations in burden of comorbid disease, income deprivation or previous admissions within 30 days.

### Care pathways

#### Admission

Of the 163 128 emergency admissions, 37 979 (23.3%) occurred on weekends; 111 782 were patients who attended the ED through self-referral or ambulance transfer, while 38 342 were referrals from GPs, the bed bureau, or outpatient clinics, these patients being admitted directly to a hospital ward. Despite a virtually identical ED attendance rate (273 mean attendances per day on weekends, 271 per day on weekdays), the mean daily hospital admission rate was lower on weekends than on weekdays (91 vs 120). The reduction in admissions on weekends was mainly attributable to fewer referrals from GPs and the bed bureau (8 per day on weekends, 25 per day on weekdays) and from outpatient clinics (2 vs 7) rather than self-presentations and emergency ambulance transfers (74 per day on weekends, 77 per day on weekdays). There was no evidence of a secular trend between 2012 and 2015.

#### Intensive care transfers

During the 4-year period, 5360 (3.3%) patients were transferred to intensive care within 24 hours of an emergency admission. There were more ICU24 patients on weekends (4.2%) than on weekdays (3.0%) (table 1). Of these, more patients were transferred to the ICU directly from the ED on weekends than on weekdays (2.9 per weekend day, 76.1% vs 2.4 per weekday, 65.3%).

**Table 1:** Pathway and population characteristics

	Weekend	Weekday	Overall	P values*﻿
ED attendances (mean/day; % admitted)	113 913 (273; 27.2%)	282 422 (271; 28.6%)	396 335 (271; 28.2%)	0.018; <0.001
Admissions (mean/day)	37 979 (91)	125 149 (120)	163 128 (112)	<0.001
Admission route (mean/day)				
ED	31 023 (74)	80 759 (77)	111 782 (77)	<0.001
GP+bed bureau	3197 (8)	26 596 (25)	29 793 (20)	<0.001
Outpatient clinic	859 (2)	7690 (7)	8549 (6)	<0.001
Other providers	2900 (7)	10 104 (10)	13 004 (9)	<0.001
ICU transfers (n (%) of weekend/weekday total admits; % of weekend/weekday ICU24 cases)				
n (% total); n/day	1579 (4.2%)	3781 (3.0%)	5360 (3.3%)	<0.001
Direct	1202 (3.2%; 76.1%)	2469 (2%; 65.3%)	3671 (2.3%; 68.5%)	<0.001; <0.001
Indirect	377 (1%; 23.9%)	1312 (1%; 34.7%)	1689 (1%; 31.5%)	0.348; <0.001
Sex				
Male	19 360 (51.0%)	62 375 (49.8%)	81 735 (50.1%)	<0.001
Female	18 619 (49.0%)	62 774 (50.2%)	81 393 (49.9%)	<0.001
Age (years)				
Median (IQR)	60 (40–78)	60 (42–77)	60 (41–77)	0.295
Mean (SD)	58.2 (22.239)	58.5 (21.393)	58.4 (21.593)	0.056
Ethnicity				
White	28 947 (76.2%)	96 339 (77.0%)	1 25 286 (76.8%)	0.005
Asian	4681 (12.3%)	15 515 (12.4%)	20 196 (12.4%)	0.856
Black	1622 (4.3%)	5322 (4.3%)	6944 (4.3%)	1.000
Mixed	507 (1.3%)	1511 (1.2%)	2018 (1.2%)	0.859
Other	1253 (3.3%)	3294 (2.6%)	4547 (2.8%)	0.201
Not stated	969 (2.6%)	3168 (2.5%)	4137 (2.5%)	0.862
Charlson Comorbidity Index				
0	18 626 (49.0%)	61 393 (49.1%)	80 019 (49.1%)	0.733
1–5	6716 (17.7%)	21 646 (17.3%)	28 362 (17.4%)	0.072
>5	12 637 (33.3%)	42 110 (33.6%)	54 747 (33.6%)	0.278
Income deprivation decile				
Median (IQR)	4 (1–6)	4 (1–6)	4 (1–6)	0.858
Mean (SD)	4.2 (2.788)	4.2 (2.770)	4.2 (2.774)	0.621
Previous admissions†				
0	33 330 (87.8%)	110 028 (87.9%)	143 358 (87.9%)	0.601
1	3943 (10.4%)	13 071 (10.4%)	17 014 (10.4%)	1.000
2	575 (1.5%)	1696 (1.4%)	2271 (1.4%)	0.150
≥3	131 (0.3%)	354 (0.3%)	485 (0.3%)	1.000
NEWS24 value				
Median (IQR)	1 (0–3)	1 (0–2)	1 (0–2)	<0.001
Mean (SD)	1.8 (2.049)	1.7 (1.969)	1.7 (1.989)	0.008
Length of stay (days)				
Median (IQR)	3 (1–8)	2 (1–7)	2 (1–7)	<0.001
Mean (SD)	7.1 (13.129)	6.9 (13.248)	7.0 (13.221)	0.008
Z-LOS (%)	6529 (17.2)	27 390 (21.9)	33 919 (20.8)	<0.001

ED, emergency department; GP, general practitioner; ICU, intensive care unit; NEWS, National Early Warning Score; Z-LOS, zero length of stay.

*Comparison between weekend and weekday.

†Within 30 days prior to the current admission.

#### Length of stay

The mean and median length of hospital stay were longer for weekend emergency admissions than weekdays (7.1 and 3.0 days, respectively, on weekends compared with 6.9 and 2.0 days on weekdays (p=0.008 for mean and p<0.001 for median)). Z-LOS patients (discharged or died before midnight on day of admission) constituted 20.8% of admissions (table 1); the proportion of such patients on weekends (17.2%) was lower than on weekdays (21.9%) but with a higher mortality (online supplementary ESM table 3).

### National Early Warning Score

**Figure 1:**
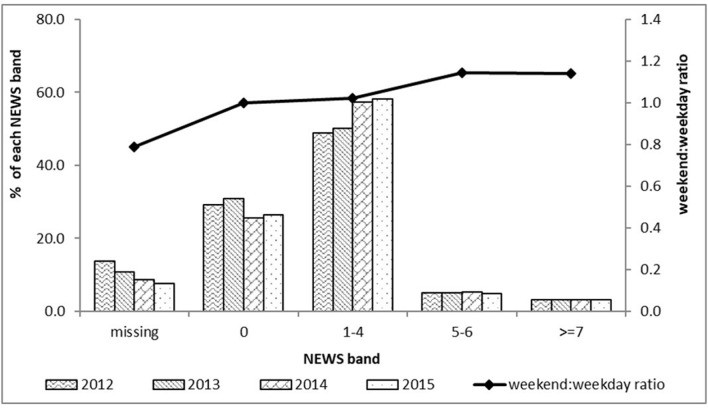
NEWS distribution in first 24 hours of admission and ratio of the distribution of each NEWS band weekend:weekday, 2012–2015. NEWS, National Early Warning Score.

Complete sets of variables permitting calculation of a NEWS within the first 24 hours were available for 90% of admissions overall. There was a secular trend for improvement in documentation of vital signs permitting calculation of NEWS from 86% of admissions in 2012 to 92% in 2015 (figure 1). NEWS completion rates were slightly higher for weekend (91.7%) admissions than weekday (89.5%) (figure 1 and online supplementary ESM table 3). NEWS could be calculated within 4 hours of admission for 72.5% of patients and within 8 hours for 83% (online supplementary ESM table 3). As expected and described above, vital signs for calculation of NEWS were more likely to be missing in ICU24 patients (63.4%) than in patients not transferred to ICU within 24 hours (8.2%), particularly for direct ICU transfers from the ED (83.5% missing NEWS) rather than indirect via another hospital location (19.8%) (online supplementary ESM table 3).

Weekend admissions had a higher NEWS than weekday (table 1) (mean NEWS 1.8 weekend, 1.7 weekday, p=0.008), and a greater proportion of weekend admissions were in the higher NEWS bands than weekday admissions (figure 1). In the non-ICU24 population, increasing NEWS values were positively associated with increasing age and length of hospital stay (online supplementary ESM table 4).

### Mortality rates and mortality adjustment

The analyses of in-hospital and 30-day mortality produced substantively similar results. Here we focus on 30-day mortality, relegating analyses of in-hospital mortality to the electronic supplementary material (online supplementary ESM tables 1 and 2; figure 4a,b).

Crude mortality rates are summarised in table 2. There were 8397 deaths within 30 days out of the 163 128 available admissions, an overall mortality rate of 5.1%. Among weekend admissions the rate was higher (5.6% compared with 5.0% on weekdays), with a (crude, unadjusted) WEOR of 1.13 (95% CI 1.08 to 1.19). Thirty days after admission, survival curves show the differences in weekend/weekday mortality rates starting to appear after 2 days (online supplementary ESM figure 5).

**Table 2:** Thirty-day crude mortality by weekend/weekday admission, stratified by ICU transfer and NEWS banding

Admission group		Crude mortality (%)						
		Weekend		Weekday		Total		WEOR (95% CI)
All admissions		2140/37 979	(5.6)	6257/125 149	(5.0)	8397/163 128	(5.1)	1.13 (1.08 to 1.19)
ICU24		275/1579	(17.4)	673/3781	(17.8)	948/5360	(17.7)	0.97 (0.83 to 1.14)
Non-ICU24	NEWS ≥7	390/1257	(31.0)	1033/3627	(28.5)	1423/4884	(29.1)	1.13 (0.98 to 1.30)
	NEWS 5–6	325/2091	(15.5)	862/6008	(14.3)	1187/8099	(14.7)	1.10 (0.96 to 1.26)
	NEWS 1–4	895/20 535	(4.4)	2895/66 297	(4.4)	3790/86 832	(4.4)	1.00 (0.92 to 1.08)
	NEWS=0	164/10 467	(1.6)	532/34 578	(1.5)	696/45 045	(1.5)	1.02 (0.85 to 1.22)
	NEWS missing	91/2050	(4.4)	262/10 858	(2.4)	353/12 908	(2.7)	1.88 (1.47 to 2.40)

ICU, intensive care unit; NEWS, National Early Warning Score; WEOR, weekend-to-weekday mortality OR.

As expected, mortality varied substantially between the strata defined by ICU transfer and NEWS banding. The rate was much higher among ICU24 admissions (17.7%) than in the non-ICU24 admissions (4.7%, p<0.001) and there was a significant upward trend in mortality with NEWS band (p<0.001) among non-ICU24 admissions where NEWS was available, ranging from 1.5% (NEWS=0) to 29.1% (NEWS≥7). Mortality for non-ICU24 admissions with missing NEWS was 2.7%, which falls within the range of the two lowest NEWS bands.

Crude (ie, unadjusted) WEORs within strata are shown in the final column of table 2. Within five out of the six risk strata (ie, ICU24 and the four non-ICU24 bands with NEWS available) the WEOR is consistent with the hypothesis that the risk stratification has eliminated the weekend effect in that each CI includes the value 1. The size of the WEOR (1.88, p<0.001) in the non-ICU24+missing NEWS group presents an anomaly. Despite the low average mortality rate (2.7%) it appears that the risk is significantly higher for weekend than for weekday admissions in this group.

**Figure 2:**
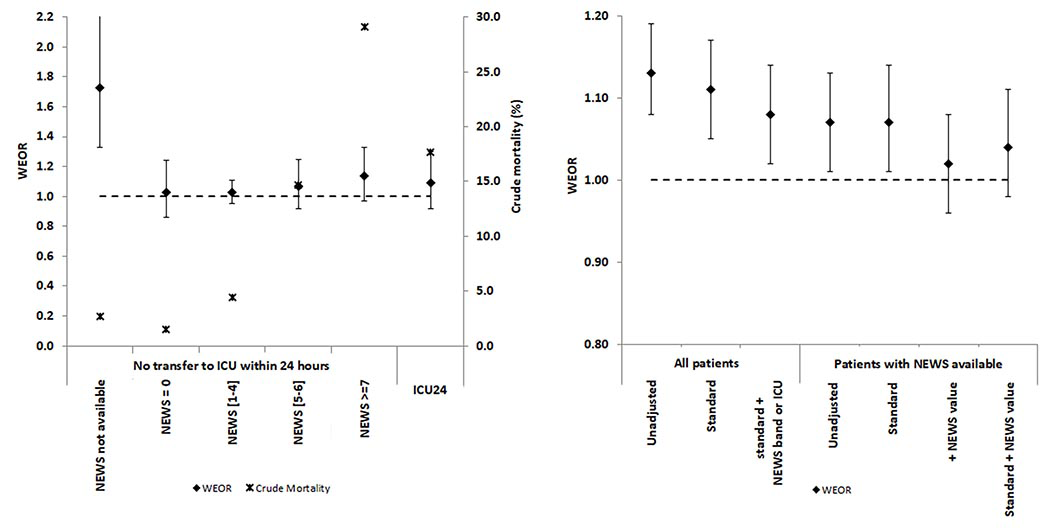
Thirty-day crude mortality (%) and adjusted weekend admission ORs (±95% CI): effect of non-availability of NEWS, increasing NEWS band and ICU transfer within 24 hours. Thirty-day weekend admission mortality ORs (±95% CI), all patients and NEWS available; *impact of adjustment variables. ICU, intensive care unit; NEWS, National Early Warning Score; WEOR, weekend-to-weekday mortality OR.

For the 146 822 (90%) admissions where NEWS could be calculated, the 30-day mortality rate was 1825/34 833=5.2% on weekends and 5506/111 989=4.9% on weekdays, with unadjusted WEOR=1.07 (95% CI 1.01 to 1.13); adjustment using the standard model had little impact (table 3 and figure 2B). Adjustment using the NEWS score alone plausibly annulled the weekend effect (WEOR=1.02, 95% CI 0.96 to 1.08), and the addition of standard adjustment variables made no useful contribution (table 3).

**Table 3:** Thirty-day crude and adjusted weekend-to-weekday mortality ORs (WEOR).

		Crude WEOR	With standard adjustment	Adjustment by NEWS* only	Standard adjustment+NEWS*
Admissions with NEWS available (n=146 822)		1.07 (1.01–1.13)	1.07 (1.01–1.14)	1.02 (0.96–1.08)	1.04 (0.98–1.11)
All admissions (n=163 128)		1.13 (1.08–1.19) †	1.11 (1.05–1.17)	(standard adjustment+ICU24/NEWS strata)	1.08 (1.02–1.14)
ICU24		0.97 (0.83–1.14) †	1.09 (0.92–1.30)		
Non-ICU24	NEWS ≥7	1.13 (0.98–1.30) †	1.14 (0.97–1.33)		
	NEWS 5–6	1.10 (0.96, 1.26) †	1.07 (0.92–1.25)		
	NEWS 1–4	1.00 (0.92, 1.08) †	1.03 (0.95–1.11)		
	NEWS=0	1.02 (0.85, 1.22) †	1.03 (0.86–1.24)		
	NEWS missing	1.88 (1.47, 2.40) †	1.73 (1.33–2.25)		

ICU, intensive care unit; NEWS, National Early Warning Score.

*NEWS included as an uncategorised covariate.

†Values from table 2.

For the full sample of admissions (including those with missing NEWS), stratified estimates of the WEOR can be calculated using the ICU24/NEWS categories, with allowance also for standard adjustment variables. The results are presented in table 3. This approach leads to a modest attenuation of the WEOR from 1.13 to 1.08 (95% CI 1.02 to 1.14) but does not appear to eliminate the weekend effect altogether, a consequence of the very high WEOR value (1.88) in the non-ICU, missing NEWS stratum.

## Discussion

In this 4-year study of weekend and weekday emergency admissions to a large university teaching hospital, we have shown that weekend admissions have a higher 30-day mortality rate which attenuates only very modestly with standard case mix adjustment, but effectively disappears when adjusted for physiological severity (NEWS) in the 90% of patients for whom complete NEWS could be calculated within 24 hours of admission. This indicates that patients admitted to hospital on weekends are sicker—more acutely ill—than those admitted on weekdays. This conclusion is supported by our other novel observations. A substantially greater proportion of weekend admissions are transferred to intensive care in the first 24 hours following admission, and more of these are transferred directly from the ED on weekends, again indicating that weekend admissions are more severely ill at hospital presentation. Importantly, we find no clinically important difference in age, gender or comorbid disease between weekend and weekday admissions: the main difference is in acuity, not chronicity.

In terms of care quality, on weekends more patients are transferred to the ICU directly from the ED, whereas on weekdays ICU referrals are more likely to follow an indirect route, suggesting the possibility of delays in patient flow on weekdays: indirect (and hence potentially delayed) admission to intensive care is associated with worse outcomes.^23^ Weekend admission NEWS completion rates are also slightly better than weekdays. These observations do not support the view that in-hospital care is of lower quality on weekends; indeed, they suggest that emergency admissions are processed more efficiently on weekends than on weekdays. Weekend admissions remain in hospital longer than those admitted on weekdays. Given the above observations it would suggest that longer stays are also an indicator of sicker weekend admissions and may not be wholly due to delayed processes on weekends.

Why should weekend admissions be more severely ill? We have shown that despite a constant daily ED attendance rate, fewer patients are admitted to hospital on weekends. The reduction in admissions is mainly attributable to a reduction in community referrals from GPs (family doctors). This supports the observation of Meacock *et al* 14 that ‘direct’ hospital admission rates are lower on weekends. Z-LOS patients constituted 20.8% of all admissions. Most Z-LOS patients are likely to be low-risk individuals who can be discharged to the community before midnight. We found fewer Z-LOS patients on weekends, and they had a higher mortality risk (online supplementary ESM table 3). The reduced number could be a consequence of difficulty in discharging frail or elderly patients back to the community on weekends. The higher mortality is also consistent with the finding that weekend admissions are more severely ill.

Taken together these findings support the hypothesis that the proximate explanation for the ‘weekend effect’ is a reduction in the denominator (fewer weekend admissions) combined with a higher mortality in the numerator (online supplementary ESM figure 1) attributable to greater severity of illness in the admitted population. To determine causation requires examination of the whole patient pathway, particularly referral patterns and the organisation of care in the community as well as hospital admission policies. Potential explanations requiring further research include the reduction in primary care services on weekends causing delayed presentation of sicker patients, or referral to hospital of patients who on weekdays might have received palliative care at home from practitioners more familiar with their circumstances. Palliative care admissions might also explain the higher weekend admission mortality risk among the non-ICU, missing NEWS group.

The strength of our study lies in the greater detail provided by the data set than could be obtained from hospital episode statistics, the duration, evaluation of an acuity dynamic measure of severity of illness (NEWS) independently of conventional measures of case mix and the exploration of pathway effects. The association of missing NEWS with mortality and with ICU admission is a novel finding likely explained by the patient pathway rather than quality of care; our study adds value to the work presented by Mohammed *et al* 20 in this respect. Uniquely, our study links differences in weekend:weekday referral and admission patterns to case mix, severity of illness, ICU admission (direct and indirect) and outcome, providing a consistent pattern across these various measures.

As with all observational studies, there are a number of weakness and limitations. This is a single-centre study raising questions about generalisability. However, the hospital serves a large and mixed population, data cover 4 years and the direction of effect is consistent with other studies using different methodologies and indirect end points. In our data set, ‘Missing NEWS’ is not a random phenomenon: it is associated with direct admissions to intensive care, bypassing the electronic data capture through which NEWS variables are recorded. It could also be associated with other factors, for example, a desire to minimise burdens in palliative care admissions. In either case it is reasonable to assume that these patients would have had a high NEWS value had this been recorded. While comorbid disease may be assessed using the Charlson Comorbidity Index, we have no specific measures of frailty. We were unable reliably to document processes of care, palliative care referral, or cardiopulmonary resuscitation, which might have provided additional explanatory value. Thus, the problems raised by patients with missing NEWS scores cannot be addressed by imputation methods which are valid only when data are ‘missing-at-random’ in the sense that the presence or absence of a measurement must not be independently associated with its numerical value.^24^ Finally, we note that hospital mortality rates bear little relationship to care quality.^25^

The implications of our study are that future research should focus on care and referral processes throughout the patient pathway. This must include care in the community, hospital admission decisions and quality of care in hospital following admission. The last of these is being addressed through the HiSLAC programme.^26^ Examining community care processes should be included in the UK policy initiative of integrating social care in the remit of the Department of Health, supported by the recent set of guidelines on integrating acute and emergency care published by the National Institute for Health and Care Excellence.^27^
