# Supplementary material for: Sicker patients account for the weekend mortality effect among adult emergency admissions to a large hospital trust
Source: BMJ Qual Saf. 2018 Oct 9;28(3):223–30. doi: 10.1136/bmjqs-2018-008219 (PMC6560459; doi:10.1136/bmjqs-2018-008219)
Supplement: Supplementary data [file bmjqs-2018-008219supp001.pdf]

ELECTRONIC SUPPLEMENTARY MATERIAL

ESM Fig 1: Mechanisms for the weekend effect

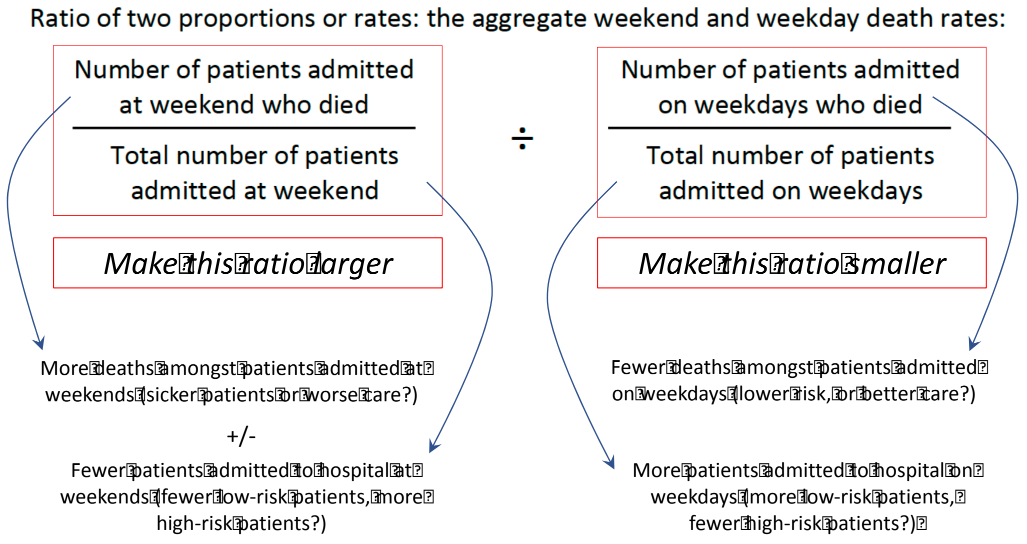

ESM Fig 2: National Early Warning Score (NEWS)*

PHYSIOLOGICAL PARAMETERS	3	2	1	0	1	2	3
Respiration Rate	≤8		9 - 11	12 - 20		21 - 24	≥25
Oxygen Saturations	≤91	92 - 93	94 - 95	≥96			
Any Supplemental Oxygen		Yes		No			
Temperature	≤35.0		35.1 - 36.0	36.1 - 38.0	38.1 - 39.0	≥39.1	
Systolic BP	≤90	91 - 100	101 - 110	111 - 219			≥220
Heart Rate	≤40		41 - 50	51 - 90	91 - 110	111 - 130	≥131
Level of Consciousness				A			V, P, or U

*The NEWS initiative flowed from the Royal College of Physicians' NEWSDIG, and was jointly developed and funded in collaboration with the Royal College of Physicians, Royal College of Nursing, National Outreach Forum and NHS Training for Innovation.

*taken from National Early Warning Score (NEWS) – Standardising the assessment of acute-illness severity in the NHS, Royal College of Physicians 2012

ESM Fig 3: The NEWS trigger system aligned to the scale of clinical risk*

NEW scores	Clinical risk
0	Low
Aggregate 1–4	
RED score* (Individual parameter scoring 3)	Medium
Aggregate 5–6	
Aggregate 7 or more	High

*taken from National Early Warning Score (NEWS) – Standardising the assessment of acute-illness severity in the NHS, Royal College of Physicians 2012

ESM Fig 4a: In-hospital crude mortality (%) and adjusted weekend admission odds ratios (± 95% CI): Effect of non-availability of NEWS, increasing NEWS band, and ICU transfer within 24 hours

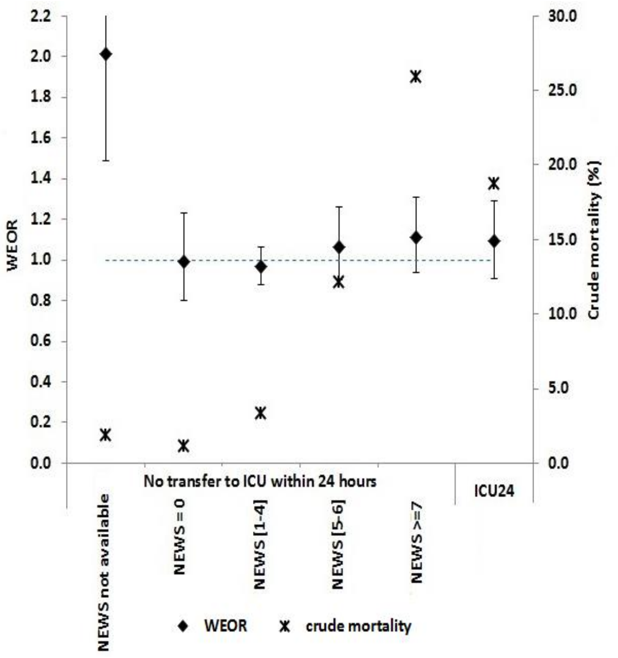

ESM Fig 4b: In-hospital weekend admission mortality odds ratios (± 95% CI), all patients and NEWS available: Impact of adjustment variables*

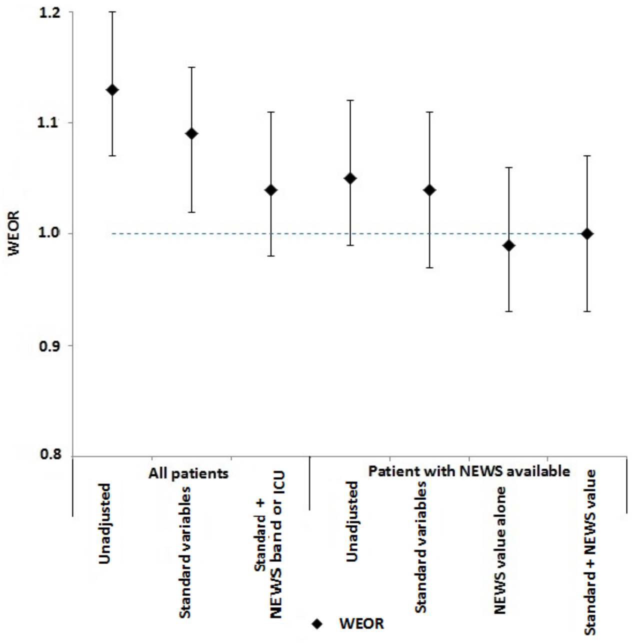

*NEWS band or ICU is status within 24 hours post admission

ESM Fig 5: 30 days post admission survival

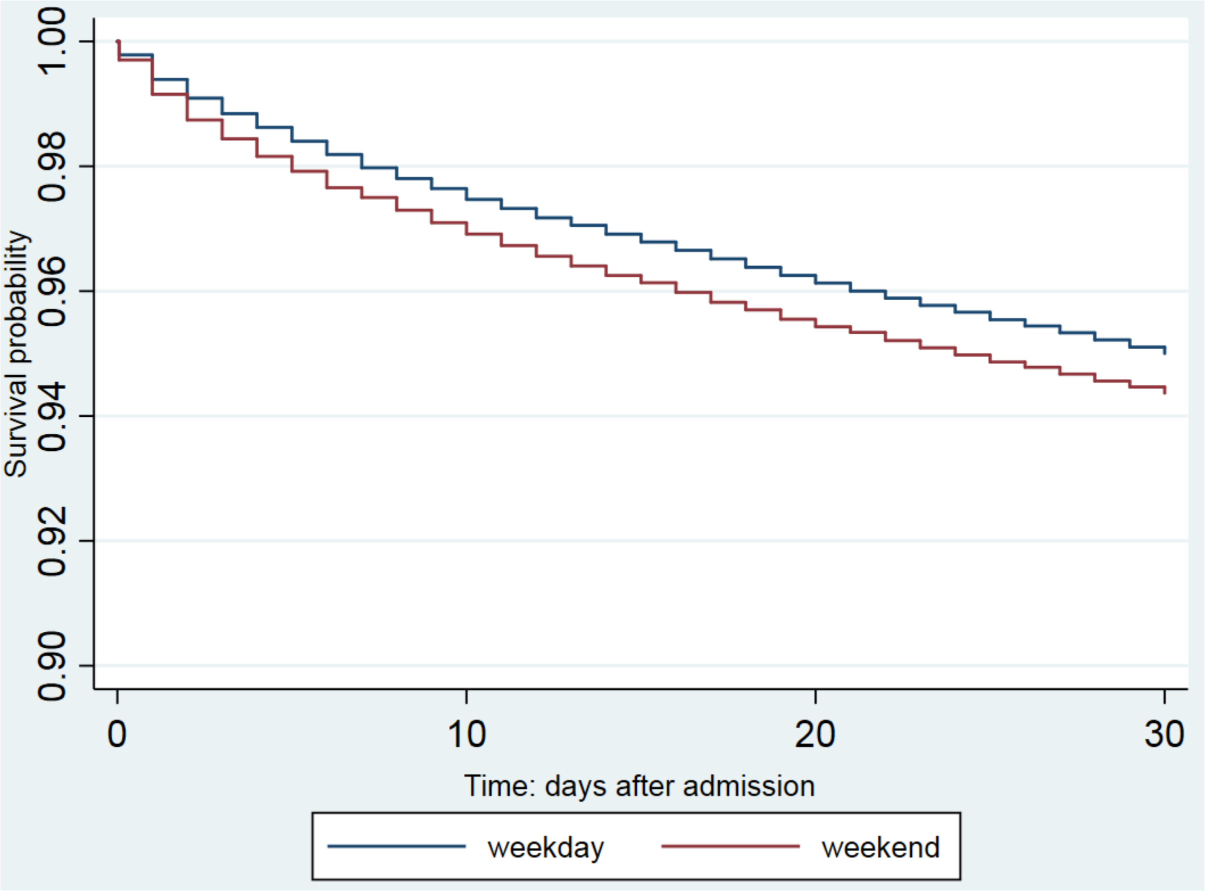

ESM Table 1. In-hospital crude mortality by weekend/weekday admission, stratified by ICU transfer and NEWS banding

Admission group		Crude Mortality (%)						
		Weekend		Weekday		Total		WEOR (95% CI)
All Admissions		1752/37979	4.6%	5116/125149	4.1%	6868/163128	4.2%	1.13 (1.07-1.20)
ICU24		291/1579	18.4%	713/3781	18.9%	1004/5360	18.7%	0.97 (0.84-1.13)
Non ICU24	NEWS >=7	347/1257	27.6%	917/3627	25.3%	1264/4884	25.9%	1.13 (0.98-1.30)
	NEWS [5-6]	270/2091	12.9%	714/6008	11.9%	984/8099	12.1%	1.10 (0.95-1.28)
	NEWS [1-4]	654/20535	3.2%	2212/66297	3.3%	2866/86832	3.3%	0.95 (0.87-1.04)
	NEWS = 0	116/10467	1.1%	384/34578	1.1%	500/45045	1.1%	1.00 (0.81-1.23)
	NEWS missing	74/2050	3.6%	176/10858	1.6%	250/12908	1.9%	2.27 (1.73-2.99)

ESM Table 2. In-hospital crude and adjusted weekend-to-weekday mortality odds ratios (WEOR).

		Crude WEOR	with Standard adjustment	Adjustment by NEWS* only	Standard adjustment + NEWS*
Admissions with NEWS available (N = 146,822)		1.05 (0.99-1.12)	1.04 (0.97-1.11)	0.99 (0.93-1.06)	1.00 (0.93-1.07)
All Admissions (N = 163,128)		1.13 (1.07-1.20) †	1.08 (1.02-1.15)	(Standard adjustment + ICU24/NEWS strata)	1.04 (0.98-1.11)
ICU24		0.97 (0.84-1.13) †	1.08 (0.91-1.29)		
Non ICU24	NEWS >=7	1.13 (0.98-1.30) †	1.12 (0.95-1.32)		
Non ICU24	NEWS [5-6]	1.10 (0.95-1.28) †	1.07 (0.90-1.26)		
Non ICU24	NEWS [1-4]	0.95 (0.87-1.04) †	0.96 (0.88-1.06)		
Non ICU24	NEWS = 0	1.00 (0.81-1.23) †	0.99 (0.80-1.23)		
Non ICU24	NEWS missing	2.27 (1.73-2.99) †	1.98 (1.47-2.67)		

*NEWS included as an uncategorised covariate

†Values from Table 2.

ESM Table 3: Case characteristics – NEWS24 status, ICU24-NEWS24 status, Z-LOS and associated mortality

	Weekend		Weekday		Overall		p_value
**Total (average per day)**	37979	(91)	125149	(120)	163128	(112)	
**NEWS24 status (%)**							
missing	3146	(8.3%)	13160	(10.5%)	16306	(10.0%)	<0.001
available	34833	(91.7%)	111989	(89.5%)	146822	(90.0%)	<0.001
**Availability of NEWS variables from time of hospital admission**							
< 4 hours	25871	(74.30%)	80605	(72.00%)	106476	(72.50%)	<0.001
< 6 hours	27859	(80.00%)	87682	(78.30%)	115541	(78.70%)	<0.001
< 8 hours	29185	(83.80%)	92488	(82.60%)	121673	(82.90%)	<0.001
< 12 hours	30918	(88.80%)	98443	(87.90%)	129361	(88.10%)	<0.001
**ICU24 transfer cases (% of weekend/weekday total; % of the category; average per day)**							
NEWS 24 missing	1096	(69.4%; 34.8%; 2.6)	2302	(60.9%; 17.5%; 2.2)	3398	(63.4%; 20.8%; 2.3)	<0.001; <0.001
NEWS24 available	483	(30.6%; 1.4%; 1.2)	1479	(39.1%; 1.3%; 1.4)	1962	(36.6%; 1.3%; 1.3)	<0.001; 0.154
Total	1579	(100.0%; 4.2%; 3.8)	3781	(100.0%; 3.0%; 3.6)	5360	(100.0%; 3.3%; 3.7)	-- ; <0.001
**No ICU24 transfer cases (% of weekend/weekday total; % of the category; average per day)**							
NEWS 24 missing	2050	(5.6%; 65.2%; 4.9)	10858	(8.9%; 82.5%; 10.4)	12908	(8.2%; 79.2%; 8.8)	<0.001; <0.001
NEWS24 available	34350	(94.4%; 98.6%; 82.4)	110510	(91.1%; 98.7%; 105.9)	144860	(91.8%; 98.7%; 99.2)	<0.001; 0.154
Total	36400	(100.0%; 95.8%; 87.3)	121368	(100.0%; 97.0%; 116.3)	157768	(100.0%; 96.7%; 108.0)	-- ; <0.001
**by NEWS band or ICU (%)**							
ICU24	1579	(4.2%)	3781	(3.0%)	5360	(3.3%)	<0.001
Non ICU24, NEWS>=7	1257	(3.3%)	3627	(2.9%)	4884	(3.0%)	<0.001
Non ICU24, NEWS [5-6]	2091	(5.5%)	6008	(4.8%)	8099	(5.0%)	<0.001
Non ICU24, NEWS [1-4]	20535	(54.1%)	66297	(53.0%)	86832	(53.2%)	<0.001
Non ICU24, NEWS = 0	10467	(27.6%)	34578	(27.6%)	45045	(27.6%)	1.000
Non ICU24, NEWS missing	2050	(5.4%)	10858	(8.7%)	12908	(7.9%)	<0.001
**Z-LOS (%)**							
ICU24	45	(2.8%)	92	(2.4%)	137	(2.6%)	0.394
Non ICU24, NEWS>=7	64	(5.1%)	191	(5.3%)	255	(5.2%)	0.784
Non ICU24, NEWS [5-6]	116	(5.5%)	351	(5.8%)	467	(5.8%)	0.611
Non ICU24, NEWS [1-4]	3073	(15.0%)	11222	(16.9%)	14295	(16.5%)	<0.001
Non ICU24, NEWS = 0	1952	(18.6%)	7652	(22.1%)	9604	(21.3%)	<0.001
Non ICU24, NEWS missing	1279	(62.4%)	7882	(72.6%)	9161	(71.0%)	<0.001
Total	6529	(17.2%)	27390	(21.9%)	33919	(20.8%)	<0.001
**Died at discharge of Z-LOS cases (%)**							
ICU24	31	(68.9%)	68	(73.9%)	99	(72.3%)	0.539
Non ICU24, NEWS>=7	41	(64.1%)	77	(40.3%)	118	(46.3%)	0.001
Non ICU24, NEWS [5-6]	8	(6.9%)	15	(4.3%)	23	(4.9%)	0.263

Non ICU24, NEWS [1-4]	2	(0.1%)	22	(0.2%)	24	(0.2%)	0.247
Non ICU24, NEWS = 0	3	(0.2%)	4	(0.1%)	7	(0.1%)	0.255
Non ICU24, NEWS missing	28	(2.2%)	73	(0.9%)	101	(1.1%)	<0.001
Total	113	(1.7%)	259	(0.9%)	372	(1.1%)	<0.001
**Direct /Indirect ICU*** (% of weekend/weekday total; % of weekend/weekday ICU24 cases)**							
direct ITU	1202	(3.2%; 76.1%)	2469	(2.0%; 65.3%)	3671	(2.3%; 68.5%)	<0.001; <0.001
indirect ITU	377	(1.0%; 23.9%)	1312	(1.0%; 34.7%)	1689	(1.0%; 31.5%)	0.348; <0.001
**Indiirect ICU by NEWS status**							
News available	303	(80.4%)	1052	(80.2%)	1355	(80.2%)	0.078
NEWS unavailable	74	(19.6%)	260	(19.8%)	334	(19.8%)	0.078
**Direct ICU by NEWS status**							
News available	180	(15.0%)	427	(17.3%)	607	(16.5%)	0.078
NEWS unavailable	1022	(85.0%)	2042	(82.7%)	3064	(83.5%)	0.078

ESM Table 4: Subgroup characteristics stratified by ICU and NEWS status within 24 hours post admission

	Weekend	Weekday	Overall	p_value*
Total (average per day)	37979 (91)	125149 (120)	163128 (112)	
**Age (years, median (IQR))**				
No ICU24, NEWS missing	51 (34-67)	53 (37-69)	53 (36-69)	<0.001
No ICU24, NEWS = 0	56 (37-76)	57 (39-74)	57 (39-74)	0.541
No ICU24, NEWS [1-4]	61 (41-78)	62 (43-78)	61 (42-78)	0.083
No ICU24, NEWS [5-6]	73 (56-83)	71 (56-83)	71 (56-83)	0.125
No ICU24, NEWS >=7	75 (62-84)	74 (62-84)	74 (62-84)	0.316
ICU24	50 (33-66)	54 (39-67)	54 (37-67)	<0.001
**Age (years, mean (SD))**				
No ICU24, NEWS missing	51.2 (20.296)	53.4 (20.116)	53.1 (20.160)	0.000
No ICU24, NEWS = 0	56.0 (22.309)	56.2 (21.261)	56.2 (21.509)	0.462
No ICU24, NEWS [1-4]	58.9 (22.350)	59.2 (21.632)	59.2 (21.805)	0.018
No ICU24, NEWS [5-6]	68.0 (19.592)	67.5 (19.065)	67.7 (19.202)	0.361
No ICU24, NEWS >=7	71.3 (17.285)	70.8 (17.308)	71.0 (17.301)	0.376
ICU24	50.3 (19.815)	53.3 (18.616)	52.4 (19.024)	<0.001
**Sex (male (%))**				
No ICU24, NEWS missing	1063 (51.8%)	5108 (47.0%)	6171 (47.8%)	<0.001
No ICU24, NEWS = 0	5464 (52.2%)	17670 (51.1%)	23134 (51.4%)	0.008
No ICU24, NEWS [1-4]	10190 (49.6%)	32664 (49.3%)	42854 (49.4%)	0.395
No ICU24, NEWS [5-6]	1005 (48.0%)	2877 (47.9%)	3882 (47.9%)	0.679
No ICU24, NEWS >=7	634 (50.4%)	1681 (46.4%)	2315 (47.4%)	0.000
ICU24	1004 (63.6%)	2375 (62.8%)	3379 (63.0%)	0.053
**Length of stay (days median (IQR))**				
No ICU24, NEWS missing	0 (0-1)	0 (0-1)	0 (0-1)	<0.001
No ICU24, NEWS = 0	2 (1-5)	2 (1-5)	2 (1-5)	0.001
No ICU24, NEWS [1-4]	3 (1-8)	3 (1-8)	3 (1-8)	0.437
No ICU24, NEWS [5-6]	6 (2-13)	6 (2-14)	6 (2-14)	0.167
No ICU24, NEWS >=7	6 (3-13)	7 (3-14)	7 (3-14)	0.012
ICU24	11 (5-23)	11 (6-24)	11 (6-24)	0.875
**Length of stay (days, mean (SD))**				
No ICU24, NEWS missing	1.8 (6.515)	1.2 (5.373)	1.3 (5.574)	<0.001
No ICU24, NEWS = 0	5.2 (10.341)	5.3 (10.767)	5.3 (10.670)	0.573
No ICU24, NEWS [1-4]	7.1 (12.983)	7.3 (12.982)	7.2 (12.982)	0.200
No ICU24, NEWS [5-6]	10.7 (14.385)	11.6 (16.657)	11.3 (16.105)	0.035
No ICU24, NEWS >=7	10.5 (13.257)	12.0 (15.925)	11.6 (15.295)	0.004
ICU24	19.0 (23.445)	19.8 (26.172)	19.5 (25.399)	0.334
**Charlson Index (%)**				
**No ICU24, NEWS missing**				
0	1273 (62.1%)	7029 (64.7%)	8302 (64.3%)	0.022
1-5	322 (15.7%)	1725 (15.9%)	2047 (15.9%)	0.838
>5	455 (22.2%)	2104 (19.4%)	2559 (19.8%)	0.003
**No ICU24, NEWS = 0**				
0	5926 (56.6%)	19550 (56.5%)	25476 (56.6%)	0.885
1-5	1747 (16.7%)	5701 (16.5%)	7448 (16.5%)	0.629
>5	2794 (26.7%)	9327 (27.0%)	12121 (26.9%)	0.571
**No ICU24, NEWS [1-4]**				
0	9630 (46.9%)	30480 (46.0%)	40110 (46.2%)	0.020
1-5	3714 (18.1%)	11699 (17.7%)	15413 (17.8%)	0.149
>5	7191 (35.0%)	24118 (36.4%)	31309 (36.1%)	<0.001
**No ICU24, NEWS [5-6]**				
0	629 (30.1%)	1722 (28.7%)	2351 (29.0%)	0.218
1-5	409 (19.6%)	1222 (20.3%)	1631 (20.1%)	0.444
>5	1053 (50.4%)	3064 (51.0%)	4117 (50.8%)	0.614
**No ICU24, NEWS >=7**				
0	315 (25.1%)	826 (22.8%)	1141 (23.4%)	0.098
1-5	285 (22.7%)	729 (20.1%)	1014 (20.8%)	0.053
>5	657 (52.3%)	2072 (57.1%)	2729 (55.9%)	0.003
ICU24				
0	853 (54.0%)	1786 (47.2%)	2639 (49.2%)	<0.001
1-5	239 (15.1%)	570 (15.1%)	809 (15.1%)	0.955
>5	487 (30.8%)	1425 (37.7%)	1912 (35.7%)	<0.001
